# Complete mitogenome mapping of potato late blight pathogen, *Phytophthora infestans* A_2_ mating type

**DOI:** 10.1080/23802359.2017.1280699

**Published:** 2017-02-16

**Authors:** Virupaksh U. Patil, G. Vanishree, Debasis Pattanayak, Sanjeev Sharma, Vinay Bhardwaj, B. P. Singh, S. K. Chakrabarti

**Affiliations:** a Division of Crop Improvement, Central Potato Research Institute (ICAR), Shimla, India;; b National Research Centre for Plant Biotechnology (NRCPB), New Delhi, India;; c Division of Plant Protection, Central Potato Research Institute (ICAR), Shimla, India;; d Central Potato Research Institute (ICAR), Shimla, India

**Keywords:** Phytophthora, mating type, haplotype mitochondria, genome, mapping

## Abstract

Complete mitochondrial genome of *Phytophthora infestans*, A_2_ mating type (MT) with a size of ≅37,767 bp was sequenced. A total of 53 protein-coding genes are predicted on both strands, including 25 *tRNA*, 2 *rRNA*, and 18 respiratory proteins. Gene order of A_2_MT was consistent with that established in A_1_, despite high level of polymorphism in both coding and non-coding regions. The mtDNA of A_2_MT was found to have 99.5% and 99.4% homology with Ia and Ib, whereas 94.7% and 94.3% with IIa and IIb, respectively. Study of repeats revealed a dinucleotide (AT)_9_ specific to A_1_ and homology of *cox1* gene sequence revealed the relationship among 50 *Phytophthora* species.

Ever since triggering the Irish famine, *Phytophthora infestans* (Mont.) has continued to wreak havoc on potato fields throughout world and its population has undergone drastic changes with new population detected, having more pathotypes, carrying new MT and being resistant to metalaxyl (Chimote et al. 2010; Arora et al. 2014). Since past two decades, it is reported to be heterothallic, resulting in high level of genetic variation and rapid evolution (Singh et al. 1994). Population displacement by genotypes with increased fitness is a recurrent event, for instance in India both MTs exists, with A_2_MT completely replacing A_1_MT in the hills and almost stabilizing, while in the plains A_1_MT has established itself (Chimote et al. 2010; Arora et al. 2014). Polymorphism at various regions of mitochondrial-genome and even complete mitochondrial-genome of *P. infestans* has been successfully employed to study origin, migration, and diversity (Hwang et al. 2014). To date mtDNA of A_1_MT have been sequenced, revealing the phylogenetic relationship among haplotype I(a&b) and II(a&b), but no efforts have been made to excavate relationship existing between the MTs(A_1_/A_2_). In the present study, complete mitochondrial-genome sequence of A_2_MT is reported and compared with A_1_MTs along with three related *Phytophthora* species.

*P. infestans* (HP10-31),^1^ belonging to haplotype-Ia (Carter et al. 1990) and A_2_MT, were isolated from late blight-infected potato fields in Shimla, placed in mid-Himalayas with wet temperate climate (31.61°N,77.10°E). The culture was grown and maintained (Caten & Jinks 1968), harvested mycelium was crushed and dispersed in pre-cooled sucrose buffer. The nuclei and mitochondria were separated by differential centrifugation (Klimczak & Prell 1984) and DNA was isolated by CTAB (Murray & Thompson 1980). The mtDNA was sheared; shotgun library was prepared (Roche-Diagnostics) and sequenced using gsFLX_Titanium (Roche-454), and 40Mb data was obtained with coverage of ≅100×. The sequence data was assembled using GS_DeNova_Assember and GS_Ref_Mapper (Roche-Diagnostics) with mitochondrial-genome of Ia as reference, yielding single mega scaffold of 37,767bp covering entire genome with 22.38% GC content. A total of 55 protein-coding genes were predicted using mVISTA, including 26 tRNA (tRNAScan-SE), 16 ribosomal proteins (RNAmmer_v1.2), 18 respiratory proteins, and an import protein *Sec-Y* (independent transport protein). None of the predicted genes possessed introns, ATG is the start codon for all the genes whereas TAA is the stop codon for all the genes except *nadh11* which has TGA (Korkmaz et al. 2014). MISA studies revealed the presence of only *Di-*and *Tri-*nucleotide repeats among all *Phytophthora* species. Two *Di-*nucleotide motifs (AT)_7_ and (AT)_6_ were found to be common in both MTs, whereas motif (AT)_15_ was found specific to A_2_MT, further exploited for differentiating MTs. The A_2_ mating type formed close cluster with haplotype Ia (0.995) and Ib (0.994) of A1 mating type in the phylogenic relationship using average nucleotide identity (ANI) (Figure 1). Comparing complete sequence of *cox1*(1479 bp) gene for 50 *Phytophthora* species using CLUSTALW revealed that *P. infestans*, *P. iranica*, *P. mirabilis*, *P. clandestina*, *P. andina*, *P. ipomeae* and *P. phaseoli* formed a single cluster indicating the close relations between species (Martin & Tooley 2003; Kroon et al. 2004). The remaining *Phytophthora* species formed a bigger cluster, whereas *P. aphanidermatum* and *P. brassicae* formed unexpectedly separate branches (Kroon et al. 2004).

**Figure 1.**
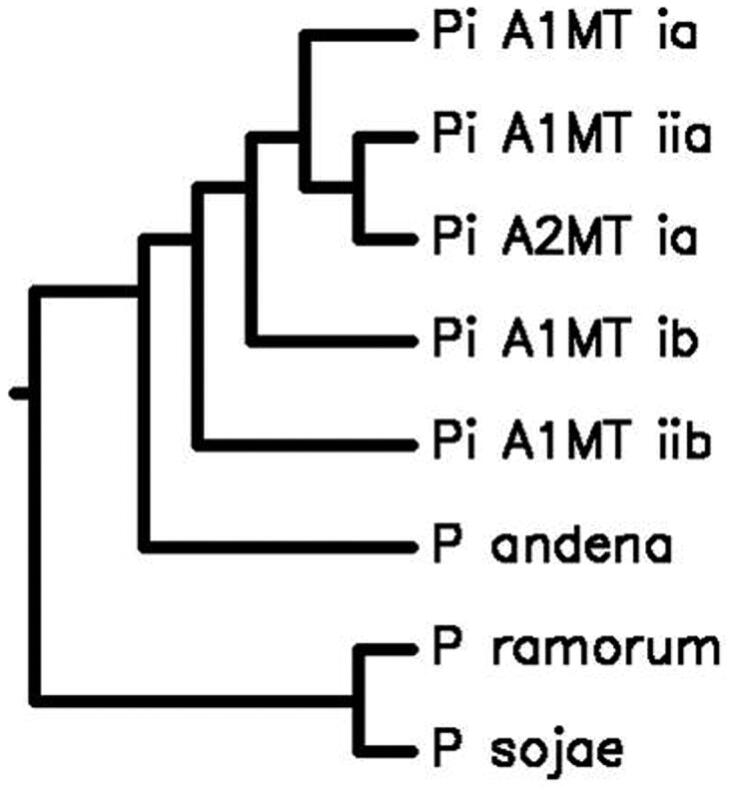
The phylogenetic tree depicting Genome-wide comparative studies of A_2_ MT with A_1_ MTs, *P. ramorum*, *P. sojae* and *P. andina* using Average Nucleotide Identity http://enve-omics.ce.gatech.edu/ani/. *P. infestans* A_1_ MT has 99.5%, 94.2%, 99.4%, and 94.3% sequence similarity with Ia (Acc. No. AY894835), IIa (Acc. No. AY898627), Ib (Acc. No. NC002387), and IIb (Acc. No. AY898628), respectively, and 98.3%, 71.5%, and 67.5% sequence similarity with *P. andina* (Acc. No. NC015619), *P. ramorum* (Acc. No. NC009384), and *P. sojae* (Acc. No. NC009385), respectively. Several nuclear and mitochondrial gene studies have shown that *P. andina* is an hybrid, with *P. infestans* as one of the parents (Kroon et al. 2004; Gómez-Alpizar et al. 2008; Haas et al. 2009; Goss et al. 2011; Blair et al. 2012; Lassiter et al. 2015) and our results strongly support the same at whole-genome level.

The high diversity found between mitochondrial-genomes of two MTs may not be only due to host specialization among the pathogens leading to evolution of novel mitochondrial lineages, but may be due to the migration dynamics and the climatic variations. The hypothesis is further supported by the increase in the GC content of the mt genome of A_2_MT. However, before firm conclusions about the genome variability between the MTs can be drawn, additional comparisons among more genotypes are needed to clarify this.

## Nucleotide sequence accession no.

Complete mitochondrial genome sequence of *P. infestans* A_2_ mating type (Haplotype Ia) is submitted to NCBI/GeneBank under the accession no. KU837230, and first draft sequence of whole genome has been submitted under Acc. No. LYVM00000000, Version: LYVM01000000.

## Note

1 The specimen *P. infestans* Phylotype Ia and A_2_ Mating Type has been submitted to gene bank for agriculturally important microbes at ICAR-Central Potato Research Institute, Shimla with No. HP10-31.

## Abbreviations

MT: Mating type
mt: Mitochondria
CTAB: Cetyltrimethylammonium bromide
ANI: Average nucleotide identity
MISA: MIcroSAtellite identification tool
