# Correction to: Patil et al., Complete mitogenome mapping of potato late blight pathogen, Phytophthora infestans A2 mating type

**DOI:** 10.1080/23802359.2018.1438827

**Published:** 2019-07-29

Patil, VU, Vanishree G, Pattanayak D, Sharma S, Bhardwaj V, Singh BP, Chakrabarti SK. (2017). Complete mitogenome mapping of potato late blight pathogen, Phytophthora infestans A2 mating type. *Mitochondrial DNA Part B: Resources.* https://doi.org/10.1080/23802359.2017.1280699.

This corrigendum is published to include the following acknowledgement of the contributions made to the first draft sequence of the whole genome, submitted under Accession no. LYVM00000000, Version: LYVM01000000:

